# Markers of Oxidative Stress in Adolescents with Skeletal Class II Division 1 Malocclusion

**Published:** 2018-12

**Authors:** Vesna OBRADOVIC, Ivan SREJOVIC, Vladimir ZIVKOVIC, Tamara NIKOLIC, Jovana JEREMIC, Dragan DJURIC, Vladimir JAKOVLJEVIC

**Affiliations:** 1. Dept. of Dentistry, Faculty of Medical Sciences, University of Kragujevac, Kragujevac, Serbia; 2. Dept. of Physiology, Faculty of Medical Sciences, University of Kragujevac, Kragujevac, Serbia; 3. Dept. Pharmacy, Faculty of Medical Sciences, University of Kragujevac, Kragujevac, Serbia; 4. Institute of Medical Physiology “Richard Burian”, School of Medicine, University of Belgrade, Belgrade, Serbia; 5. Dept. of Human Pathology, IM Sechenov 1st Moscow State Medical University, Moscow, Russia

## Dear Editor-in-Chief

The association between increased production of reactive oxygen species (ROS) and jaw dysfunction is still poorly known especially in youth population. The present study was aimed to assess different parameters of oxidative stress in malocclusion class II division 1 of adolescents (1–3).

This case-control study included 80 female and male adolescents (between 18 and 21 yr old) with permanent dentition with or without malocclusion, examined at the Institute of Dentistry, Kragujevac, Serbia. According to degree of malocclusion, all participants were divided into the following groups: 1. control group - included healthy participants with overjet 2–4 mm; 2. Group A - included participants who had class II division 1 malocclusion with overjet 5–7 mm; 3. Group B - included participants who had class II division 1 malocclusion with overjet 8–10 mm; 4. Group C - included participants who had class II division 1 malocclusion with overjet > 10 mm. Malocclusion evaluation and classifications are based on the relationship of the mesiobuccal cusp of the maxillary first molar and the buccal groove of the mandibular first molara as previously described (4). Collection of unstimulated whole saliva was performed (5). From samples of unstimulated saliva following oxidative stress parameters were determined: index of lipid peroxidation (measured as TBARS), nitrites (NO_2_^−^), superoxide anion radical (O_2_^−^), and hydrogen peroxide (H_2_O_2_) (6).

All patients before inclusion in the study signed an informed consent. The study was approved by the Committee of Ethics of the local institution (Faculty of Medical Sciences, University of Kragujevac) and in compliance with ethical standards.

Values of all estimated pro-oxidant markers were statistically higher in presence of malocclusion comparing to control (*P*<0.05, *P*<0.01) (Table 1). Values of TBARS, H_2_O_2_ and O_2_^−^ were statistically higher in groups B and C comparing to control (*P*<0.01), while the values of nitrites were significantly higher in groups A and C comparing to control (*P*<0.05) (Table 1). Present study may help in elucidation of involvement of free radicals in pathogenesis of jaw dysfunction. Our results have shown strong positive correlation between increased values of oxidative stress markers and degree of malocclusion, suggesting predictive role of these markers in following course of disease. These findings can be of clinical interest in terms of implementation of antioxidants as adjuvant therapy in persons with malocclusion.

**Table 1:** Index of lipid peroxidation (TBARS), nitrites (NO_2_^−^), superoxide anion radical (O_2_^−^) and hydrogen peroxide (H_2_O_2_) values presented as mean±SD

***Variable***	***Control group***	***Group A***	***Group B***	***Group C***
TBARS (μmol/ml)	3.53 ± 0.52	3.40 ± 0.57	7.10 ± 0.54	6.78 ± 0.54
NO_2^−^_ (μmol/ml)	14.45 ± 1.81	19.25 ± 1.32	17.70 ± 2.60	30.70 ± 6.04
O_2^−^_ (nmol/ml)	2.38 ± 0.62	2.41 ± 0.84	6.71 ± 0.81	6.62 ± 0.98
H_2_O_2_ (nmol/ml)	0.47 ± 0.06	0.44 ± 0.08	2.38 ± 0.07	1.93 ± 0.15
